# Microwave-Assisted Improved Extraction and Purification of Anticancer Nimbolide from *Azadirachta indica* (Neem) Leaves

**DOI:** 10.3390/molecules25122913

**Published:** 2020-06-24

**Authors:** Panawan Suttiarporn, Vachira Choommongkol

**Affiliations:** 1 Faculty of Science, Energy and Environment, King Mongkut’s University of Technology North Bangkok, Rayong campus 21120, Thailand; panawan.s@sciee.kmutnb.ac.th; 2 Department of Chemistry, Faculty of Science, Maejo University, Chiang Mai 50290, Thailand

**Keywords:** nimbolide, *Azadirachta indica*, optimization, response surface methodology, microwave-assisted extraction

## Abstract

Nimbolide, a limonoid present in leaves of the neem tree (*Azadirachta indica*), is an anticancer compound against a panel of human cancer cell lines. The rapid process of extraction and purification of the nimbolide from the leaves of neem tree through microwave-assisted extraction (MAE) coupled with a chromatographic technique was accomplished. The crude with a maximum content of nimbolide could be recovered from neem leaves through MAE. By using three-factors, three-level Box–Behnken design of response surface methodology (RSM), the optimal conditions for nimbolide extraction (*R*^2^ = 0.9019) were solid/liquid ratio 1:16 g/mL, microwave power 280 W, and extraction time 22 min. The enriched extract was further purified by a preparative thin-layer chromatography (PTLC), where nimbolide was obtained as 0.0336 g (0.67% yield, purity over 98%) with ethyl acetate/hexane = 4:6 in 3.0 h. Structural elucidation was performed through spectroscopic techniques, including FT-IR, ^1^H, and ^13^C-NMR. This method was simple and had a good potential for the purification of bioactive compounds from a natural product.

## 1. Introduction

*Azadirachta indica* A. Juss belongs to the family Meliaceae, and is commonly known as “neem”. The neem tree, an extraordinary plant, is reported to be a tree that can solve global problems, by the US National Academy of Science [1]. The neem is cultivated in the Indian subcontinent and other regions of the world in at least 30 countries ranging from Asia to Africa, and as far as America [2]. Almost all parts of the plant, including leaves, flowers, fruits, seeds, and bark are extensively used in traditional medicine for treating various human diseases [3,4].

**Figure 1:**
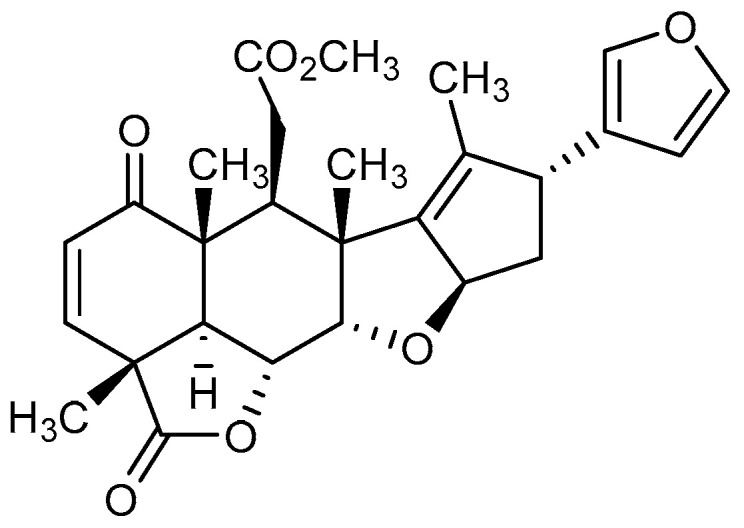
Chemical structure of nimbolide.

Neem is one of the richest sources of biologically active secondary metabolites, particularly tetranortriterpenoids or limonoids, such as azadirachtin, nimbolide, nimbin, salannin, nimbidin, and gedunin [5,6]. Among the limonoid compounds, nimbolide, as shown in Figure 1, is the major active constituent present in the leaves of *Azadirachta indica* [7]. The nimbolide was reported to be an “anticancer compound” as it showed promising anticancer activities against a variety of human cancer types, including breast cancer, cervical cancer, choriocarcinoma, colon cancer, pancreatic cancer, and prostate cancer [8,9]. Moreover, the compound shows therapeutic properties like antimalarial activity [3], antibacterial activity [10], antifeedant [11], and antioxidant [12]. 

Recently, there has been an increasing demand for plant bioactive substances that can be used as functional ingredients in pharmaceutical and nutraceutical products due to their multitarget therapeutic properties, with minimal or no undesirable side-effects [13]. There is a rise in interest in the potential use of the nimbolide constituent as an anti-cancer drug [8].

Selection of a proper extraction method to isolate bioactive compounds should be a key consideration due to sample preparation, the first step in the analysis of any medicinal plant study, and the important role it plays on the final outcome. Biologically active substances from the neem tree are mainly extracted using traditional techniques, such as maceration [14] and heating reflux extraction [15]. However, these methods are time-consuming and laborious. The supercritical fluid extraction, as an alternative method, is also reported for the separation of nimbolide from neem leaves (*Azadirachta indica)* [16]. A new approach, microwave-assisted extraction (MAE), is a powerful technique for isolating bioactive substances from a medicinal plant and it has a number of advantages, such as a higher extraction rate, supreme product quality at a lower cost, requiring considerably less time, and consuming a smaller amount of solvent [17]. MAE was successfully used in extraction protocols for the effective isolation of limonoid, azadirachtin [18], and neem oil [19], from neem.

However, the extraction efficiency of biologically active compounds using the MAE process is influenced by various extraction conditions, such as extraction time, microwave power, the ratio of material-to-solvent, among others, and their effects might be either independent or interactive [20]. To overcome this problem, response surface methodology (RSM), a powerful multivariate statistical and mathematical tool was applied for optimizing the conditions of a complex experimental process. RSM has been successfully utilized for developing and optimizing various extraction processes from plants, to yield bioactive compounds like 1,8-cineole [21], zeaxanthin and lutein [22], total phenolic compounds and flavonoids [23], and polysaccharide [24].

To the best of our knowledge, there are no studies that have focused on the optimization of the MAE procedure using RSM and the systematic study for sequence extraction and purification of nimbolide from *Azadirachta indica* (Neem) leaves. Therefore, the objective of this study was to reveal a simple method for the isolation and purification of nimbolide from *Azadirachta indica* (Neem) leaves. The RSM method was applied to investigate the optimal MAE process for the separation of enriched nimbolide extract from *Azadirachta indica* (Neem) leaves, based on quantitative evaluation, using high-performance liquid chromatography (HPLC). After RSM optimization, the extract was further purified to obtain high purity of nimbolide using a simple preparative thin-layer chromatography (PTLC). 

## 2. Results and Discussion

### 2.1. HPLC Quantification Method Validation

The method revealed a good linearity in the range of nimbolide concentration (0.25 to 200 μg/mL). The analytical curve generated a linear equation (y = 16488x − 11479) with a determination coefficient (R^2^) of 0.9999. The LOD is the lowest detectable concentration of nimbolide in the MAE extract but it is not quantified as the exact content. Meanwhile, the LOQ, the lowest concentration of nimbolide in the MAE extract could be accurately and precisely measured. The LOD and LOQ of the analytical procedure were 0.02 μg/mL and 0.07 μg/mL, respectively. The precision data were represented by the relative standard deviation (RSD) calculated from the ratio between the standard deviation of data (n = 10) and the mean of the nimbolide content. The coefficient of variation (%RSD) value for the content of nimbolide in repeatability test was 1.46%. The RSD value between the data of two different days was 2.58%. The quantification procedure was precise, as indicated by the low RSD value (less than 5%) recommended by ANVISA [25]. The recovery value of nimbolide was 101.87% and RSD was 1.84%, which indicated that the quantification method had sufficient accuracy.

### 2.2. Screening of Single Variables

**Figure 2:**
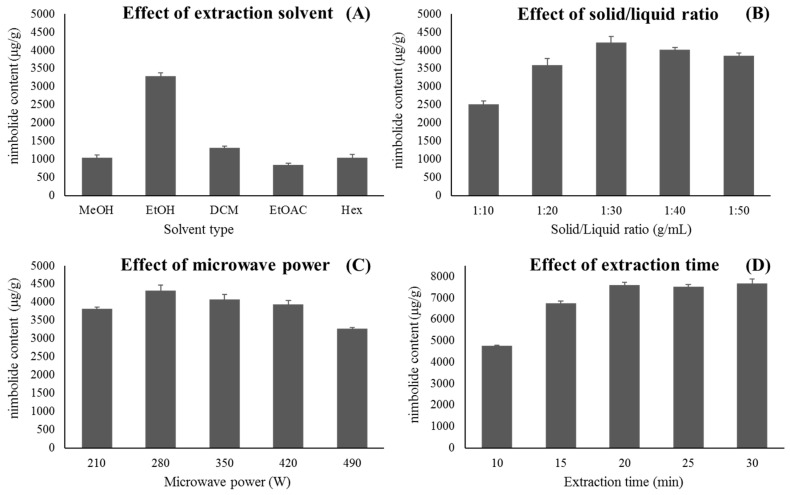
Effect of (**A**) extraction solvent, (**B**) solid/liquid ratio, (**C**) microwave power, and (**D**) extraction time on the nimbolide content.

The solvent selection is one of the most essential steps in bioactive compound extraction from plants [26] and affects microwave extraction. The high ability to absorb microwave power leads to the high ability to dissolve the compounds of interest. To yield the maximum amount of target compound, the MAE process could be performed with an appropriate solvent that has a high capacity to absorb microwave irradiation and convert the energy into heat, which depends on the solvent dielectric properties, based on the polarity of the target compound [27]. Thus, the extracted solvent (methanol, ethanol, dichloromethane, ethyl acetate, and hexane) was first selected and judged, based on extraction efficiency and operational performance. Figure 2A showed that the amount of nimbolide of *Azadirachta indica* varied from 841.07 ± 50.91 to 3.289.52 ± 85.91 μg/g weight extract/weight of dried plant material, with a descending order of ethanol (3,289.52 ± 85.91 μg/g) > dichloromethane (1,314.82 ± 49.05 μg/g) > hexane (1,042.05 ± 89.83 μg/g) > methanol (1,040.25 ± 85.06 μg/g) > ethyl acetate (841.07 ± 50.91 μg/g). The result revealed that the highest nimbolide yield was obtained by ethanol as a solvent, as compared to other solvents. This was due to an affinity between the polarity of the solvent and the compound of interest [28]. The high extraction efficiency of ethanol was due to the high solubility of the limonoid group in alcohol [29]. It was consistent with previous reports that ethanol is a good solvent system for the extraction of polyphenolic compounds from *Ocimum basilicum* [30]. Thus, ethanol was the most appropriate solvent in extraction processes, not only due to its extraction efficiency but also because of its safety for human consumption and environment.

The optimization by RSM was more effective when an appropriate range of parameters was chosen. The selection of condition ranged for the MAE, the material/solvent ratio, microwave power, and microwave time, before setting the levels of the studied factors that were also investigated by single factor experiment and the levels of the MAE studied parameters. For a single factor experiment of MAE parameters, one factor was changed in a certain range, while the remaining factors were kept constant. In Figure 2B, the solid/liquid ratio was varied from 1:10 to 10:50 g/mL, with microwave power at 210 W and extraction time at 10 min. The results indicated that the highest nimbolide content was obtained at the solid/liquid ratio 1:30 g/mL (4211.17 ± 177.11 μg/g dry weight). In liquid–solid extraction, a higher volume of extraction solvent would increase the number of target compounds until equilibrium was reached [31]. Moreover, a larger volume of solvent would cause more absorption of microwave irradiation, to ensure rapid heating up, leading to an enhanced extraction efficiency [32]. However, at an equilibrium state, where the amount of target analyte in the material was exhausted, the higher volume of the solvent would not promote the nimbolide content any longer [31]. Hence, 1:10–1:50 g/mL was selected as an appropriate range of solid/liquid ratio for further optimization.

In Figure 2C, microwave power was surveyed from 210–490 W with a solid/liquid ratio at 1:30 g/mL and extraction time at 10 min. As the irradiation increased from 210–280 W, the nimbolide content increased from 3811.98 ± 47.68 to 4323.95 ± 153.15 μg/g dry weight. By continuing to increase the microwave power, the content of the compound was reduced. The microwave power and thermal effect for the extraction process were interrelated, as a high microwave power increased the temperature in the system [33]. However, high temperature might lead to its degradation due to the thermally labile property of nimbolide in limonoid group [29]. Therefore, 210–490 W was chosen for subsequent experimental design by the Box–Behnken design (BBD).

Finally, the effect of extraction time on the content of nimbolide was studied in the range of 10–30 min with a solid/liquid ratio at 1:30 g/mL 280 W and a microwave power at 210 W (Figure 2D). The results showed that, when the extraction time increased from 10 to 20 min, the nimbolide content increased from 4758.44 ± 41.29 to 7600.86 ± 135.37 μg/g dry weight. By continuing to increase irradiation time to 30 min, the nimbolide content increased slightly to 7670.93 ± 209.98 μg/g dry weight. However, the different nimbolide contents that were achieved at 20, 25, and 30 min were not statistically significant. The prolonged time-period past a certain point, did not affect the nimbolide content, due to an eventual equilibrium between the material solute and solvent [34]. Therefore, 10–30 min was selected for further studies.

### 2.3. Response Surface Model Fitting

To achieve the highest content of the bioactive nimbolide compound, the Box–Behnken design (BBD) was utilized to further optimize the MAE parameters, including the solid/liquid ratio (1:10–1:50 g/mL), microwave power (210–490 W), and extraction time (10–30 min). The BBD was chosen, as it required a fewer number of runs. Table 1 presented the treatments with the encoded levels of variables and the experimental results of the nimbolide content. Fifteen experiments were designated with random 12 factorial experiments and 3 zero-point tests, to estimate the errors. The investigated response of 15 varied MAE conditions was analyzed through HPLC. Regression analysis of the nimbolide prediction study was employed to fit full second-degree polynomial model, including linear (X_1_, X_2_ and X_3_), quadratic ($X_{1}^{2}$, $X_{2}^{2}$ and $X_{3}^{2}$), and interaction (X_1_X_2,_ X_1_X_3_ and X_2_X_3_) at 5% level of significance. The analysis of variance (ANOVA) results for the regression equation are presented in Table 2. The model sufficiently fitted the data because due to the low *p*-value (p< 0.05), high *F*-values, and no significant lack of fit (p> 0.05). Furthermore, the model coefficients of determination (R^2^) value for the model was 90.19%, which was higher than 80% (>0.80), indicating that more than 90.19% of the response variability could be described by the experimental data. 

Moreover, the quadratic term of the microwave power (X_2_) and the extraction time (X_3_) also had a significant influence, indicating that the interrelation between the nimbolide content response variable and the MAE test variable was not linear. Thus, the developed model could be used for the MAE process optimization. The relationship between the response and the MAE independent variables was generated into the second-order polynomial Equation (1), as follows:

(1) $$Y=5300+35X_{1}-436X_{2}+94X_{3}-516X_{1}^{2}-1316X_{2}^{2}-1055X_{3}^{2}+1375X_{1}X_{2}-640X_{1}X_{3}+265X_{2}X_{3}$$

where Y is the nimbolide content, X_1_ is the solid/liquid ratio, X_2_ is the microwave power, and X_3_ is the extraction time

### 2.4. Influence and Optimization of the MAE Parameters on the Nimbolide Content

**Figure 3:**
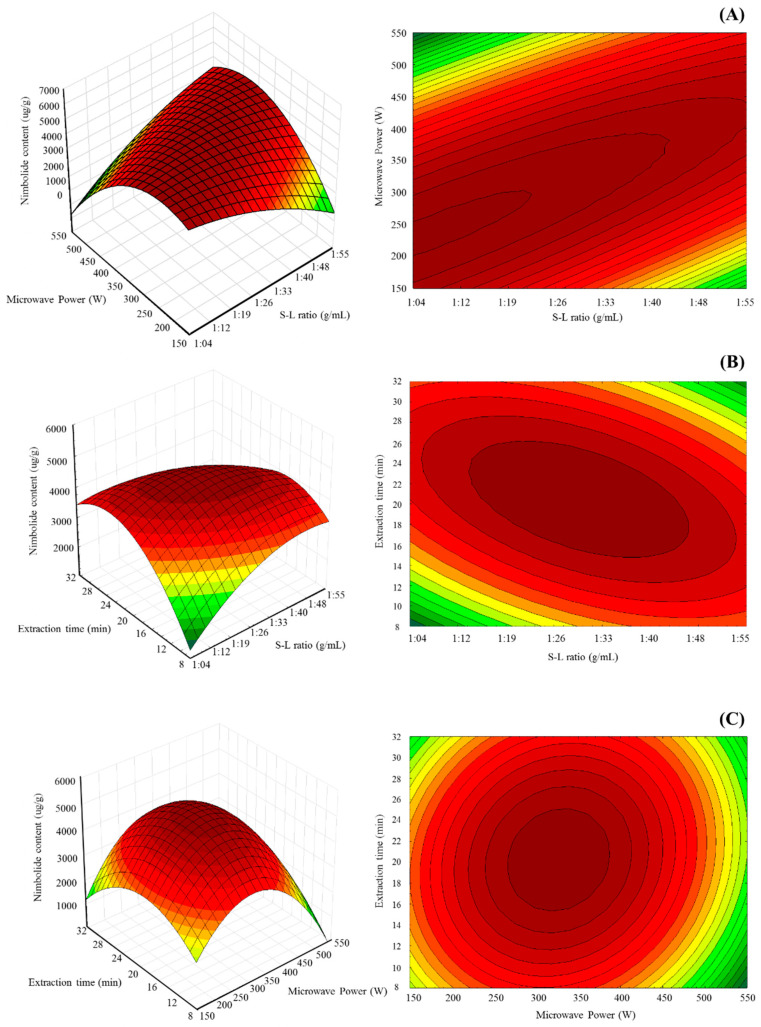
Three-dimensional and contour plots showing the effect of (**A**) solid/liquid ratio (X_1_) and microwave power (X_2_); (**B**) solid/liquid ratio (X_1_) and extraction time (X_3_); and (**C**) microwave power (X_2_) and extraction time (X_3_).

The three-dimensional response surface and two-dimensional contour plots shown in Figure 3 were used for not only investigating the optimum values but also for visualizing the effects of the mutual interactions of extraction parameters on the nimbolide content response. The interaction between the solid/liquid ratio (X_1_) and the microwave power (X_2_) had a statistically significant effect (*p* < 0.05) on the nimbolide content. The nimbolide yield significantly increased with increasing microwave power from 210–280 W, while a further increase in microwave power led to a decrease in the nimbolide content. On the other hand, at a given microwave power, nimbolide yield increased while increasing the extraction time to about 22 min. The increments in the nimbolide yield occurred due to the interaction between microwave power and extraction time. The negative influence of microwave power on nimbolide yield was due to the rapid temperature increase, as excessive irradiation could cause thermal decomposition, while the positive influence could accelerate destruction of the plant cells [35]. The maximum response value was obtained by showing the response surface plots and contour plots as the red area. The optimum selected MAE conditions were a solid/liquid ratio of 1:16 g/mL, microwave power 280 W, and an extraction time of 22 min. The maximum nimbolide content of 7046.00 μg/g dry weight was forecasted by the second-order polynomial model for enriching the nimbolide extraction. Determination of optimum conditions and the predicted value was based on desirability 0.9174.

### 2.5. Verification of the Predictive Model

To validate the predicted nimbolide content, the actual experiment of the microwave-assisted extraction was performed under optimal conditions. The results of the validated MAE extraction conditions (Table 3) showed that the actual content of nimbolide was closed to those predicted by the models with 2.80% error. This confirmed that the constructed response model was adequate in predicting the nimbolide content.

### 2.6. Purification, Determination, and Characterization of Nimbolide

Neem leaf powder (5.00 g) was extracted by MAE using the optimum selected MAE conditions. The solution was filtered and evaporated to dryness. The crude extract was dissolved with dichloromethane (DCM) to give the DCM crude extract. The crude product was successfully purified by preparative thin-layer chromatography (PTLC; silica gel), using EtOAc/hexane = 4:6 as an eluent to give a nimbolide-enriched fraction, which was further subjected to repeated purification by PTLC, to afford a high yield of nimbolide (0.0336 g, 0.67% yield). A good separation of nimbolide from other compounds occurred in 3.0 h. with microwave-assisted improved extraction, combined with a simple chromatographic technique. In comparison to conventional extraction methods, there was a considerable improvement of the nimbolide yield, which was consistent with previous reports (Table 4), as microwave irradiation can cause explosion of neem leaf structures inside the sample, releasing the nimbolide to the environment. However, Dai, J. et al. suggested that it could be carried out only in plant materials possessing relatively weak microstructures [18]. Moreover, RSM was supported to achieve the maximum nimbolide yield obtained from the MAE procedure.

## 3. Materials and Methods

### 3.1. Materials and Apparatus

Fresh leaves of neem (*Azadirachta indica* A. Juss) were collected from the Sansai, Chiang Mai Province in Thailand in February 2016. The plant was identified from the CMUB Herbarium, Chiang Mai University (Specimen voucher No. 39898). The leaves were air-dried and ground with a commercial grinder and stored at room temperature, until further analysis.

The solvents used for extraction in commercial grade (methanol, ethanol, ethyl acetate, dichloromethane, and hexane) were distilled before use. Nimbolide (CAS: 25990-37-8) was purchased from Sigma-Aldrich (St. Louis, MO, USA). Water and methanol in HPLC grade were purchased from RCI labscan (Bangkok, Thailand).

### 3.2. Microwave-Assisted Extraction (MAE) Procedure of the Enriched Nimbolide

MAE experiments were performed using an Electrolux EME2024MW system (Electrolux, Bangkok, Thailand) equipped with a reflux apparatus. Powder of neem leaf was accurately weighed, placed into a sampling flask, and operated with MAE, using different solvent and different MAE conditions. The filtered solutions were diluted 10-fold, with a suitable solvent, and were filtered through 0.45-µm pore nylon syringe filter before analysis using a high-performance liquid chromatography.

### 3.3. Quantification of Nimbolide Content Using HPLC Analysis and Method Validation

Quantitative analysis of nimbolide in neem leaves extracts was performed by PerkinElmer Flexar^tm^ HPLC equipped with photodiode array detector (PAD) detector (PerkinElmer, Waltham, MA, USA). The separation was performed on a reversed-phase Brownlee C18 column (4.6 mm, 250 mm, 5 µm, PerkinElmer, Waltham, MA, USA) at 35 °C. A mobile phase was a mixture of methanol and water in a ratio of 70:30 (*v*/*v*) used in an isocratic elution system. The flow rate of mobile phase was set at 1 mL/min. The injection volume was 10 µL. The detector wavelength of nimbolide was monitored at 217 nm. Peak identification was done by comparison of retention times of standard compound. Standard calibration curves were prepared by serially diluting the stock solutions of nimbolide in ethanol to yield concentrations of 0.25–200 µg/mL, with a correlation coefficient (R^2^) of 0.9999. All controlled instrument and data analysis/processing were performed via the PerkinElmer Chromera^@^ CDS software.

HPLC quantification of nimbolide was validated for linearity, LOD, LOQ, precision, and accuracy. The linearity was evaluated by the external calibration curve of nimbolide at seven concentration levels of the nimbolide standard (0.25, 0.50, 1.00, 5.00, 12.50, 25.00, 50.00, 100.00, and 200.00 μg/mL). The analysis was carried out in triplicate, for each test concentration, and subjected to the HPLC system. The analytical curve was fitted by linear regression and the determination coefficients were calculated. The limit of detection (LOD) and limit of quantification (LOQ) were investigated based on the standard deviation (SD) of the intercept with *y*-axis and the slope of calibration (S) of the nimbolide standard curve. Repeatability (intraday) and intermediate precision (interday) were performed. The relative standard deviation (RSD) was calculated, obtained from ten injections. The accuracy of the quantification procedure was evaluated by the recovery analysis.

### 3.4. Screening of Single Variables

Various parameters had an effect on the extraction process. The extraction factors that influenced the MAE process were solvent nature, extraction time, microwave power, and material/solvent ratio. Among these parameters, the extraction solvent was optimized as an initial step. Selection of solvents; to evaluate the solvent efficiency, methanol (MeOH), ethanol (EtOH), dichloromethane (DCM), ethyl acetate (EtOAc), and hexane (Hex), were employed as the extraction solvent, based on extraction efficiency and operational performance as an initial step. Furthermore, an appropriate condition ranges of the MAE; extraction time, microwave power, and solid/liquid ratio were studied by a single factor experiment. For the MAE single factor experiment, one factor was varied in a certain range while all other factors were kept constant. The extraction parameters were solid/liquid ratio (1:10, 1:20, 1:30, 1:40, and 1:50 g/mL), microwave power (210, 280, 350, 420, and 490 W), and extraction time (10, 15, 20, 25, and 30 min). The nimbolide content was expressed as mean ± standard deviation (SD) for three replicates. The experiments were investigated for establishing an appropriate range of independent variables in RSM.

### 3.5. Experimental Design

Operational parameters—solid/liquid ratio (X_1_), microwave power (X_2_), and extraction time (X_3_) under different MAE conditions were optimized by the maximized nimbolide content, using a response surface designed experiment combined with a Box–Behnken design (BBD). The input independent variables were coded at three levels (−1, 0, +1). The encoded levels of the variables used in the experimental design are presented in Table 5. The complete design contained 15 experiment points with 3 replications at the center. Regression analysis was performed on the data obtained by triplicate analysis for each dependent variable, using the Minitab statistics software (trial version 18, Minitab Inc., State College, PA, USA). Experimental data were fitted for maximum nimbolide content, using a second-order polynomial expressed by Equation (2), as follows:

(2) $$Y=\beta_{0}+\sum_{i=1}^{3}\beta_{i}X_{i}+\sum_{i=1}^{3}\beta_{ii}X_{i}^{2}+\sum_{i=1}^{2}\sum_{j=i+1}^{3}\beta_{ij}X_{i}X_{j}+\varepsilon$$

where “Y” is the nimbolide response; X_i_ represents the independent variables; the terms X_i_X_j_ and X_i_^2^ denote the quadratic and interaction terms, respectively; “β_0_” is the equation parameters for the constant term, “β_i_” is the linear regression coefficients, “β_ii_” is the quadratic regression coefficient, “β_ij_” is the interaction regression coefficient, and “ε” is the random error.

The data from the BBD model was also applied for prediction of the optimum extraction parameters through response surface analysis. The optimum conditions settings that would yield the maximum nimbolide content, their combined desirability, and prediction of the nimbolide content was generated by the Minitab software optimizer. Three-dimensional and contour plots were constructed using the Statistica program (Trial version 10.0, Statsoft Inc., Tulsa, OK, USA).

### 3.6. Verification of the Predictive Model

The adequacy of the optimized experimental MAE conditions in the MAE process were verified by reproducing the experiment in three replicates with the achieved optimal parameters. The actual nimbolide yield was validated by comparing the predicted value with the experimental value. 

### 3.7. Purification Process and Identification of Nimbolide

**Figure 4:**
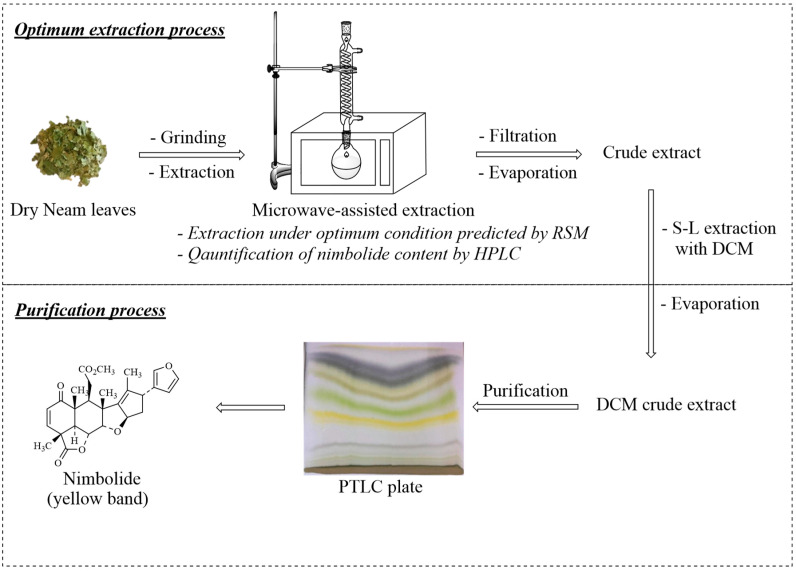
The extraction and purification process of nimbolide.

Nimbolide extraction was examined with MAE, using the optimum-selected MAE conditions. The solution was filtered and evaporated to dryness. The crude extract was re-extracted in a solid–liquid extraction process with dichloromethane (DCM), to obtain the DCM crude extract. The crude product was purified by preparative thin-layer chromatography (PTLC) plates, using EtOAc/hexane (4:6) as an eluent (Figure 4). The purification process using the PTLC plates was conducted using silica gel (silica gel 60 PF_254_, Merck, Darmstadt, Germany). The spectroscopy technique was recorded by the melting points (Gallenkamp, Manchester, UK). The Fourier transform infrared (FT-IR) spectrum was recorded on a PerkinElmer (model Spectrum RX I, Waltham, MA, USA) in reciprocal centimeters (cm^−1^). The ^1^H and ^13^C NMR experiments were performed by spectrometers (model DRX 400 MHz, Bruker, Billerica, MA) in CDCl_3_. Chemical shifts were represented as *δ*-values in parts per million (ppm), downfield from tetramethyl silane (TMS: *δ* 0.00) and relative to residue CHCl_3_ as the internal reference (^1^H: *δ* 7.26, ^13^C: *δ* 77.00), and the coupling constants (*J* values) were reported in Hertz (Hz). The peak multiplicities were represented as singlet (s), doublet (d), triplet (t), doublet of doublets (dd), and doublet of a triplet (dt). The structure elucidation of nimbolide was achieved according to their spectral data, including FT-IR, ^1^H NMR, and ^13^C NMR. The final structures of nimbolide were established by comparing the spectroscopic analysis of the standard, combined with the previous report data.

**Figure 5:**
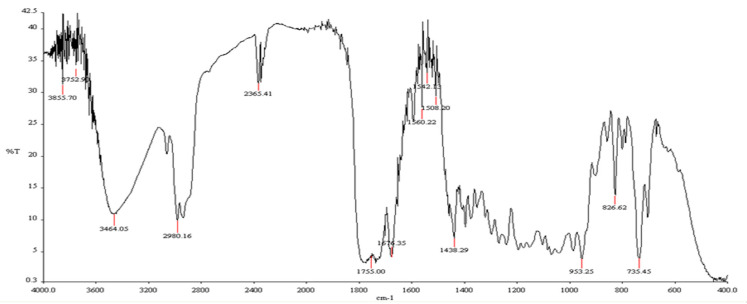
FT–IR spectrum data of nimbolide (isolated).

**Figure 6:**
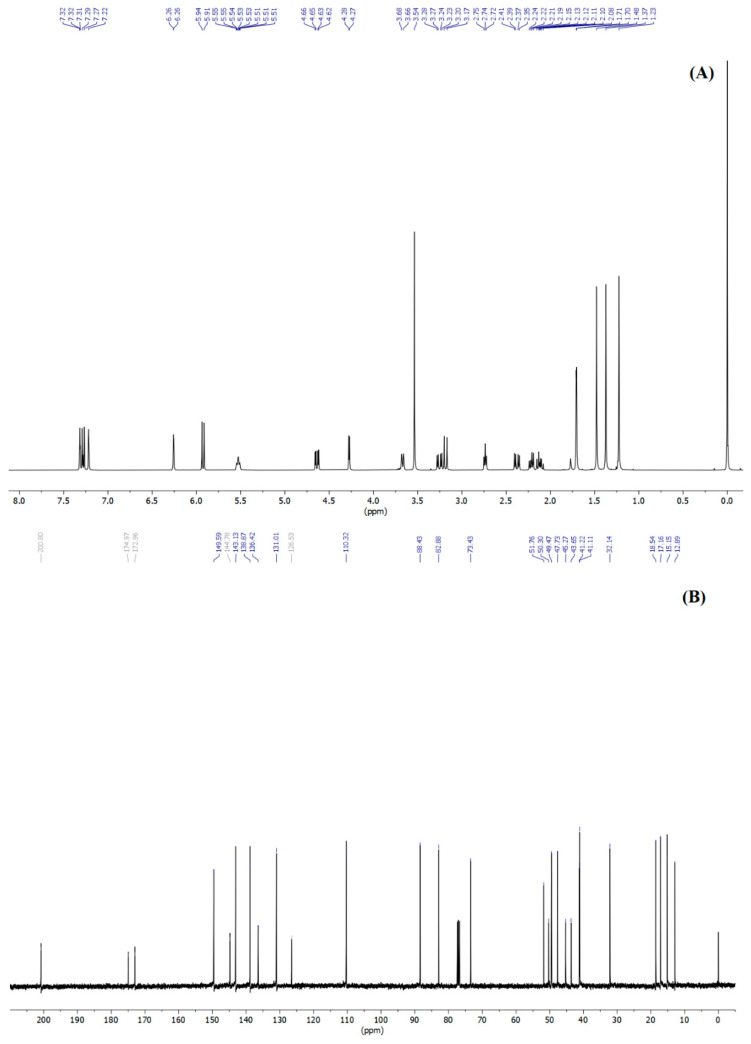
(**A**) ^1^H- and (**B**) ^13^C-NMR spectrum of nimbolide (isolated).

The characterization of nimbolide (isolated) used the IR and NMR technique. Figure 5 shows the IR spectra of the nimbolide and the chemical structure of nimbolide. Figure 6 shows ^1^H- and ^13^C-NMR spectrum of nimbolide. The main characterization list of nimbolide was as follows: yellow solids, m.p. 120.8–122.3 °C, FT-IR Spectrum (KBr); (λ max, cm^−1^): 3050, 2980, 1775, 1725, 1675, 1438, 953. ^1^H-NMR (400 MHz, CDCl_3_, δ, ppm, *J*/Hz): 1.23 (s, 3H), 1.37 (s, 3H), 1.48 (s, 3H), 1.71 (d, *J* = 1.8 Hz, 3H), 2.12 (dt, *J* = 12.1, 8.4 Hz, 1H), 2.22 (dd, *J* = 12.1, 6.7 Hz, 1H), 2.38 (dd, *J* = 16.2, 5.8 Hz, 1H), 2.74 (t, *J* = 5.6 Hz, 1H), 3.18 (d, *J* = 12.5 Hz, 1H), 3.25 (dd, *J* = 16.2, 5.3 Hz, 1H), 3.54 (s, 3H), 3.67 (d, *J* = 8.2 Hz, 1H), 4.27 (d, *J* = 3.7 Hz, 1H), 4.64 (dd, *J* = 12.5, 3.7 Hz, 1H), 5.53 (tp, *J* = 5.6, 1.8 Hz, 1H), 5.93 (d, *J* = 9.7 Hz, 1H), 6.26 (dd, *J* = 1.9, 0.9 Hz, 1H), 7.22 (s, 1H), 7.28 (d, *J* = 9.7 Hz, 1H), 7.32 (t, *J* = 1.7 Hz, 1H). ^13^C-NMR (100 MHz, CDCl_3_, δ, ppm): 12.89, 15.15, 17.16, 18.54, 32.14, 41.11, 41.22, 43.65, 45.27, 47.73, 49.47, 50.30, 51.76, 73.43, 82.88, 88.43, 110.32, 126.53, 131.01, 136.42, 138.87, 143.13, 144.78, 149.59, 172.96, 174.97, 200.80. The spectroscopic data of nimbolide (isolated) were in agreement with those of the nimbolide standard and previously reported data [36].

## 4. Conclusions

Microwave-assisted extraction was accomplished by the extraction of the enriched nimbolide product from *Azadirachta indica* A. Juss. An optimization strategy using response surface methodology based on the Box–Behnken design was successfully applied to optimize extraction with the following parameters—solid/liquid ratio 1:16 g/mL, microwave power 280 W, and extraction time 22 min. Under the optimal MAE conditions, the extraction that showed the highest nimbolide yield was 7.243 mg/g dry weight. The enriched nimbolide crude extract obtained from MAE could simply be isolated by preparative thin-layer chromatography (PTLC) with EtOAc/hexane = 4:6. The 0.0336 g of nimbolide was obtained from 5.00 g of the neem leaf sample by purifying over 98% within 3.0 h. These results revealed a new method to improve the processes of extraction and purification of nimbolide, which were simple, fast, and efficient. This procedure has a good potential for the preparation of an anticancer compound from a medicinal plant for pharmaceutical applications.

## Figures and Tables

**Table 1:** The Box–Behnken experimental design with the encoded microwave-assisted extraction (MAE) conditions and the analytically obtained values of the nimbolide content.

Std. Order	X_1_ Solid/Liquid Ratio (g/mL)	X_2_ Microwave Power (W)	X_3_ Extraction Time (min)	Nimbolide Content (ug/g DW)
12	1:30	490	30	3517.25
2	1:50	210	20	2858.00
9	1:30	210	10	2869.30
7	1:10	350	30	4058.00
1	1:10	210	20	5786.00
8	1:50	350	30	3092.88
10	1:30	490	10	2395.62
11	1:30	210	30	2932.52
6	1:50	350	10	4589.84
14	1:30	350	20	5620.12
3	1:10	490	20	1237.01
4	1:50	490	20	3809.53
15	1:30	350	20	5069.62
5	1:10	350	10	2993.05
13	1:30	350	20	5208.87

**Table 2:** Analysis of variance (ANOVA) for the response surface quadratic model of the nimbolide content.

Source	Sum of Squares	Degrees of Freedom	Mean Square	*F*-Value	*p*-Value
Model	21429495	9	2381055	5.11	0.044
Linear	1599763	3	533254	1.14	0.417
X_1_	9535	1	9535	0.02	0.892
X_2_	1519380	1	1519380	3.26	0.131
X_3_	70847	1	70847	0.15	0.713
Square	10344886	3	3448295	7.39	0.028
$X_{1}^{2}$	1162319	1	1162319	2.49	0.175
$X_{2}^{2}$	6392946	1	6392946	13.71	0.014
$X_{3}^{2}$	4109850	1	4109850	8.81	0.031
2-Way Interaction	9484847	3	3161616	6.78	0.033
X_1_*X_2_	7563935	1	7563935	16.22	0.010
X_1_*X_3_	1640850	1	1640850	3.52	0.120
X_2_*X_3_	280062	1	280062	0.60	0.473
Lack-of-Fit	2167721	3	722574	8.82	0.104
Pure Error	163856	2	81928		
Total	23761073	14			
R^2^	90.19	R^2^ (adj)	72.52		

**Table 3:** Verification of the predicted value at the optimal extraction condition.

Variables and Response	Predicted Value	Actual Result
X_1_ solid/liquid ratio (g/L)	1:16.45	1:16
X_2_ Microwave power (W)	280.71	280
X_3_ Time (min)	21.91	22
Nimbolide (mg/g dry weight)	7.046	7.243 ± 0.150
Nimbolide isolated (mg/g dry weight)	-	6.720 ± 0.500

**Table 4:** The content of the isolated nimbolide obtained from several extraction methods.

Material	Extractions Technique	Purification Technique	Nimbolide Isolated (g)	%Yield	Author
Dried-leaves (5 g)	optimal condition MAE (Ethanol)	Silica gel (PTLC)	0.0336 g	0.67	This study
Dried-leaves (500g)	Reflux (Soxhlet apparatus in DCM)	Silica gel (CC)	0.133 g	0.027	Kigodi, P. G. et al. [36]
Dried-leaves (545 g)	Reflux (Soxhlet apparatus in hexane)	Silica gel (CC)	0.716 g	0.13	Nair, M. S. et al. [37]
Dried-leaves (500 g)	Maceration (acetone)	Silica gel (CC)	0.45 g	0.09	Dhanya, S. et al. [38]

**Table 5:** The factors and levels of the Box–Behnken design of the microwave-assisted extraction process of nimbolide from neem.

Independent Variable	Level		
	−1	0	+1
**X_1_** Solid/Liquid ratio (g/mL)	1:10	1:30	1:50
**X_2_** Microwave power (W)	210	350	490
**X_3_** Extraction time (min)	10	20	30
